# Expanding the CRISPR/Cas genome-editing scope in *Xenopus tropicalis*

**DOI:** 10.1186/s13578-022-00841-3

**Published:** 2022-07-08

**Authors:** Zhaoying Shi, Hao Jiang, Guanghui Liu, Songyuan Shi, Xuan Zhang, Yonglong Chen

**Affiliations:** 1 Harbin Institute of Technology, Harbin, 150001 Heilongjiang China; 2 Guangdong Provincial Key Laboratory of Cell Microenvironment and Disease Research, Shenzhen Key Laboratory of Cell Microenvironment, Department of Biology, School of Life Sciences, Southern University of Science and Technology, Shenzhen, 518055 Guangdong China

**Keywords:** SaCas9, KKH SaCas9, LbCas12a, Fragment deletion, *Xenopus tropicalis*

## Abstract

**Background:**

The true diploid frog, *Xenopus tropicalis* (*X. tropicalis*) is an excellent genetic model organism. To date, the CRISPR/Cas-mediated genome editing methods established in this species are mostly based on SpCas9 that requires the stringent NGG protospacer-adjacent motif (PAM) for target recognition, which limits its genome editing scope. Thus, it is highly desirable to circumvent this limitation.

**Results:**

Through one-cell stage injection of Cas/gRNAs into *X. tropicalis* embryos, we evaluated the mutagenic efficiency of 8 different Cas variants using T7EI assay, Sanger DNA sequencing, or deep sequencing. Our data indicate that SaCas9 and KKH SaCas9 are highly effective in frogs, which could be used for direct phenotyping in G0 embryos. In contrast, VQR Cas9, xCas9 3.7, SpG Cas9, and SpRY Cas9 were ineffective in *X. tropicalis* embryos and no activity was detected for iSpyMac Cas9. We also found that LbCas12a/crRNA RNP complexes with paired crRNAs efficiently induced small fragment deletions in *X. tropicalis* embryos.

**Conclusion:**

SaCas9 and KKH SaCas9 are robust genome editing tools in *X. tropicalis* embryos. LbCas12a/crRNA RNP complexes are useful for inducing DNA fragment deletions in frog embryos. These tools expand the CRISPR/Cas genome editing scope in *X. tropicalis* and increase the flexibility for various genome editing applications in frogs*.*

**Supplementary Information:**

The online version contains supplementary material available at 10.1186/s13578-022-00841-3.

## Background

In the past decade, *Streptococcus pyogenes* Cas9 (SpCas9) has been widely used for genome editing in various species. The clustered regularly interspaced short palindromic repeats (CRISPR)/SpCas9 system requires a NGG PAM at the target site, which obviously limits its editing scope in any given genome [1–3]. To circumvent this limitation, several approaches have been taken. First, a number of Cas9 orthologues from various bacterial genera recognizing different PAMs have been identified and harnessed for genome editing, such as *Staphylococcus aureus* Cas9 (SaCas9) with the NNGRRT PAM [4], *Neisseria meningitidis* Cas9 (NmCas9) with the NNAGAAW PAM [5], *Streptococcus thermophilus* Cas9 (St1Cas9 and St3Cas9) with NNAGAAW and NGGNG PAMs, respectively [6], and *Campylobacter jejuni* Cas9 (CjCas9) with NNNVRYAC and NNNNRYAC PAM [7]. Second, upon directed evolution of the Cas9 PAM-interacting (PI) domain, various Cas9 variants with altered PAM preferences have been generated, such as VQR SpCas9 (NGA), VRQR SpCas9 (NGCG), xCas9 3.7 (NG, NNG, NGG, GAA, GAT, and CAA), SpG Cas9 (NGN), SpRY Cas9 (NRN and NYN), and KKH SaCas9 (NNNRRT) [8–11]. By replacing the PI domain of SpCas9 with that of *Streptococcus macacae* Cas9 (SmacCas9), the further engineered iSpyMac Cas9 hybrid can target all adenine dinucleotide (NAA) PAM sequences [12]. Third, members of Class 2 and Type V CRISPR/Cas, such as Cas12a (originally called Cpf1), has also been found to have gene editing capability in some species. Unlike Cas9, Cas12a is a single RNA-guided endonuclease lacking trans-activating crRNA (tracrRNA), which recognizes a short T-rich PAM and induces a staggered DNA double stranded break with a 4–5 nt 5′ overhang. LbCas12a and AsCas12a can be adapted for genome editing in mammalian cells, frog and zebrafish [13–15]. Engineering of Type V-B CRISPR/Cas system member, Cas12b from *Bacillus hisashii* (BhCas12b), called BhCas12b v4 that utilizes VTTV PAM located at the 5’ end of the target DNA sequence, facilitates robust genome editing in human cell lines and in primary human T cells ex vivo [16]. Recently, the mRNA-active ErCas12a has been reported effective in human cells and zebrafish embryos [17, 18].

The true diploid frog *X. tropicalis* is an excellent genetic model for both developmental genetics studies and human disease modeling [19]. To date, except for one report on the application of LbCas12a in *X. tropicalis* [14], the Cas9-mediated targeted gene disruption and integration methods as well as single base editing methods that we and others have established in *X. tropicalis* are exclusively based on SpCas9 [20–25]. Thus, it is highly desirable to expand the CRISPR/Cas genome editing scope in this species.

In this study, we analyzed mutagenic activity of 8 CRISPR/Cas variants in *X. tropicalis* embryos and found that SaCas9 and KKH SaCas9 are robust tools in frogs. In addition, LbCas12a/crRNA ribonucleoprotein (RNP) complexes showed high efficiency for DNA fragment deletion in *X. tropicalis* embryos. Adding of these tools definitely expands the CRISPR/Cas genome-editing scope in *X. tropicalis*.

## Results

### SaCas9 and KKH SaCas9 have robust gene editing activity in *X. tropicalis*

**Fig. 1:**
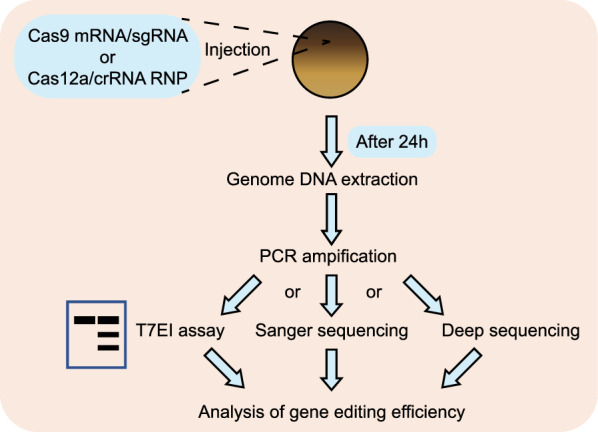
Flow chart of CRISPR Cas9 or LbCas12a disruption efficiency detection in *X. tropicalis*

**Fig. 2:**
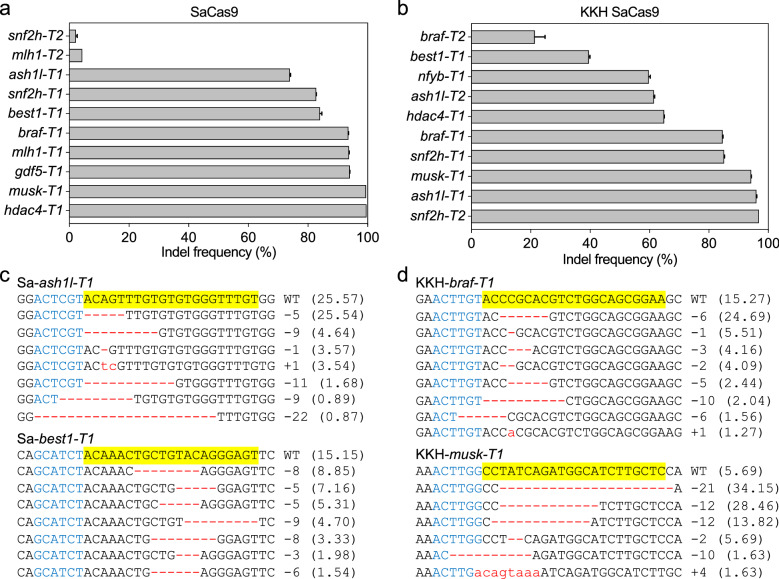
SaCas9 and KKH SaCas9 are efficient and robust tools for genome editing in *X. tropicalis.* **a** SaCas9 induced efficient targeted gene disruption in *X. tropicalis* embryos. **b** KKH SaCas9 induced efficient targeted gene disruption in *X. tropicalis* embryos. **c**, **d** Deep-sequencing data show some of the mutations induced by SaCas9 and KKH SaCas9. For all the panels, the wild-type sequence is shown at the top with the target site highlighted in yellow and the PAM sequence in blue text. Red dashes indicate deletions and lowercase letters in red indicate insertions or mutations. The numbers in parentheses show the percentage of this sequence in the total sequence reads. For target site designation, the gene name is in the middle, the prefixes Sa- and KKH- represent SaCas9 and KKH SaCas9, respectively. The suffixes -T1 and -T2 represent the first and the second target site designed in a given gene, respectively

SaCas9 and KKH SaCas9 have been proven effective in a number of species including zebrafish [4, 26]. To test their mutagenic effects in *X. tropicalis* embryos, for each of them, we designed 10 gRNAs targeting 9 different genes. Following the strategy illustrated in Fig. 1, we injected each individual Sa gRNA (50 pg/egg) together with either SaCas9 mRNA (300 pg/egg) or KKH SaCas9 mRNA (300 pg/egg) into fertilized eggs before the first cleavage. For each injected group, 18–24 h after injection, 60 healthy embryos were collected and divided into two replicates (each 30 embryos) for genome extraction, PCR amplicon library construction, and high-throughput DNA sequencing. The deep sequencing data indicated that vast majority of the target sites tested showed profound targeted genome-editing efficiency (Fig. 2). For SaCas9, half of the targeted sites (5/10) displayed indel formation frequency above 90% and only 2 appeared inefficient (Fig. 2a, c and Additional file 1: Fig. S1). For KKH SaCas9, the indel efficiency of 8 target sites is more than 60%, and that of other 2 is about 40% and 25%, respectively (Fig. 2b, d and Additional file 1: Fig. S2). Most indels detected are small deletions rarely exceeding 10 bp (Additional file 1: Figs. S1, S2). Taken together, these data indicate that both SaCas9 and KKH SaCas9 can effectively edit the genome in *X. tropicalis* embryos.

### VQR Cas9, SpG Cas9, SpRY Cas9, and xCas9 3.7 showed poor mutagenic activity in *X. tropicalis* embryos

**Fig. 3:**
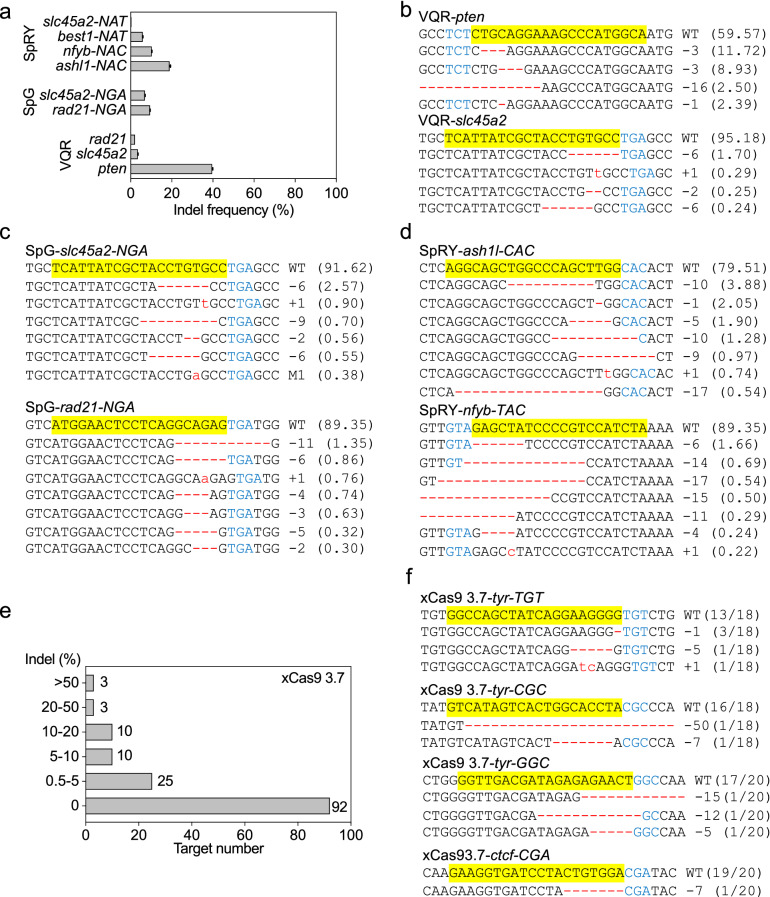
VQR Cas9, SpG Cas9, SpRY Cas9 and xCas9 3.7 have limited genome editing efficacy in *X. tropicalis*. **a** VQR Cas9, SpG Cas9, and SpRY Cas9 induced indel frequency in *X. tropicalis* embryos, as detected by deep-sequencing. **b**–**d** Some of the deep-sequencing data show the mutations induced by VQR Cas9, SpG Cas9, and SpRY Cas9. The wild-type sequence is shown at the top with the target site highlighted in yellow and the PAM sequence in blue text. Red dashes indicate deletions and lowercase letters in red indicate insertions or mutations. For all panels, the numbers in parentheses show the percentage of this sequence in the total sequence reads. **e** Summary of the xCas9 3.7 targeted genome-editing efficiency based on gray value analyses of the T7EI data. **f** DNA sequences show xCas9 3.7 induced mutations, as detected by Sanger DNA sequencing. For each panel, the wild-type sequence is shown at the top with the target site highlighted in yellow and the PAM sequence in blue text. Red dashes indicate deletions and lowercase letters in red indicate insertions or mutations. The numbers in parentheses represent the ratio of this sequence in the total colonies sequenced. For target site designation, in addition to the gene name, the name of the Cas9 variant is used as a prefix and in some cases the PAM motif is indicated as a suffix

To test more CRISPR/Cas variants for indel induction in *X. tropicalis* embryos, we designed 8, 15, 4, and 4 gRNAs for SpG Cas9, SpRY Cas9, VQR Cas9, and iSpyMac Cas9, respectively, and checked their mutagenic activity with the T7EI assay. Weak T7EI signals were detected in 2 SpG Cas9 targeting sites, 5 SpRY Cas9 sites, and 3 VQR Cas9 sites. None of the 4 iSpyMac sites displayed discernible T7EI signals (Additional file 1: Fig. S3). For the 10 T7EI positive sites, we further carried out deep sequencing analysis, which confirmed 9 T7EI signals, while the SpRY-*best1-NAT* site appeared to be a T7EI false-positive one with no indels detected by deep sequencing (Fig. 3a–d and Additional file 1: Figs. S4–S6). Gray value analysis revealed that for each site tested, the low value of the T7EI signal matched well with the corresponding low indel frequency detected by deep sequencing (Fig. 3a–d and Additional file 1: Fig. S3–S6). Large number of T7EI positive samples have routinely been confirmed by Sanger DNA sequencing in our laboratory. Thus, T7EI assay is a reliable method for detecting targeted gene disruption in our hands.

As xCas9 3.7 has a broad range of PAM compatibility including NG, NNG, NGG, GAA, GAT, and CAA, we designed 143 non-NGG PAM gRNAs targeting 6 genes (*tyr*, *ptf1a/p48*, *ctcf*, *apc*, *kcnj2*, and *bmp4*), 1 NGG PAM gRNA targeting *bace2* to cover all these PAMs and assessed their targeted genome-editing efficacy with the T7EI assay. For NGG PAM sites, we also included 3 gRNAs targeting *apc*-163, *apc*-1714, and *tbx5*-237, which had been proven effective for SpCas9 [24]. Weak T7EI signals were detected at 51 non-NGG sites and 2 NGG sites. Gray value analysis revealed that editing efficiency for most of the positive ones was about 5%. Only 3 sites displayed efficiency slightly over 50% (Fig. 3e and Additional file 1: Figs. S7, S8a). We selected 4 non-NGG sites and 2 NGG sites with different T7EI signal intensities and carried out a Sanger DNA sequencing verification. The indel frequency detected by Sanger sequencing confirmed the T7EI signals (Fig. 3f and Additional file 1: Figs. S7, S8c). For NGG sites, the editing efficiency of xCas9 3.7 is much lower than that of SpCas9 (Additional file 1: Fig. S8, and [24]). Collectively, these data indicate that VQR Cas9, SpG Cas9, SpRY Cas9, and xCas9 3.7 do have certain gene editing efficacy with low efficiency in *X. tropicalis.* But they cannot serve as robust gene editing tools in frogs.

### LbCas12a/crRNA RNP complexes are effective for DNA fragment deletion in *X. tropicalis*

**Fig. 4:**
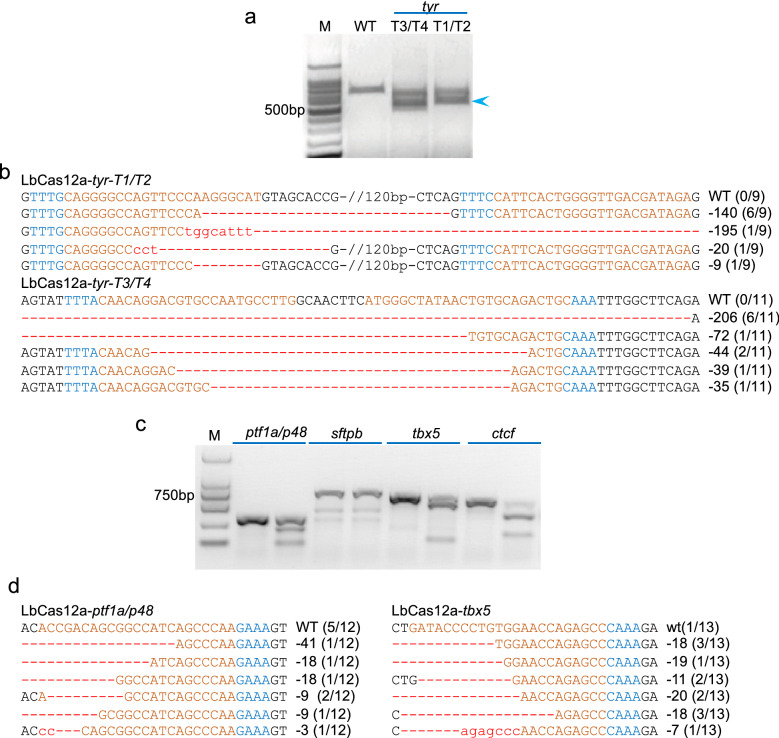
LbCas12a/crRNA RNP complex is an efficient and robust tool for short DNA fragment deletion and targeted gene disruption in *X. tropicalis*. **a** PCR assay data show segmental deletions induced by LbCas12a/crRNA RNP complex with paired crRNAs at the *tyr* locus of *X. tropicalis* embryos. M, DNA molecular weight marker. crRNAs T1 pairs with T2 and T3 pairs with T4. **b** Sanger DNA sequencing data show segmental deletions as well as indels induced by LbCas12a/crRNA RNP complex with paired crRNAs at the *tyr* locus. For both panels, the wild-type sequence is shown at the top with the target sites in red and the PAM sequence in blue. In the upper panel, 120 bp wild-type sequences were not shown. Red dashes indicate deletions and lowercase letters in red indicate insertions or mutations. The numbers in parentheses represent the ratio of this sequence in the total colonies sequenced. **c** T7EI assay data show targeted mutations induced by *ptf1a/p48*, *sftpb*, *tbx5*, or *ctcf* crRNAs and the LbCas12a RNP complexes. M, DNA molecular weight marker. **d** Sanger DNA sequencing data show targeted mutations induced by *ptf1a/p48*, or *tbx5* crRNAs and the LbCas12a RNP complexes. For both panels, the wild-type sequence is shown at the top with the target sites in red and the PAM sequence in blue. Red dashes indicate deletions and lowercase letters in red indicate mutations. The numbers in parentheses indicate the ratio of this sequence in the total colonies sequenced

Targeted genome DNA fragment deletion is key to establish animal models of human genetic disorders and to study the function of lncRNA, miRNA, cis-regulatory elements, as well as functionally uncharacterized genome regions. To test the activity of the LbCas12a/crRNA RNP complexes in inducing segmental deletion in *X. tropicalis*, we designed two pairs of crRNAs targeting two fragments on the *tyr* locus. For both pairs, gray value analysis of the PCR products on the agarose gel showed that the quantity of the fragments with deletions was roughly the same as that of wild-type fragments, which was confirmed by Sanger DNA sequencing, revealing deletion efficiency of about 50% (Fig. 4a, b). We further designed 4 crRNAs targeting 4 genes (*ptf1a/p48*, *sftpb*, *tbx5* and *ctcf*) and tested the mutagenic activity of the LbCas12a/crRNA RNP complexes with the T7EI assay. The data obtained indicate that 3 sites displayed editing efficiency above 50%, of which 2 were further confirmed by Sanger DNA sequencing (Fig. 4c, d). Most indels induced by the LbCas12a/crRNA RNP complexes are deletions that are obviously longer than those induced by SaCas9 or KKH SaCas9 (Fig. 4d and Additional file 1: Figs. S1, S2). Together, these data indicate that LbCas12a/crRNA RNP complexes are highly effective for DNA fragment deletion and indeed robust for gene editing in *X. tropicalis*.

### Phenotyping G0 embryos mutagenized by SaCas9 and KKH SaCas9

**Fig. 5:**
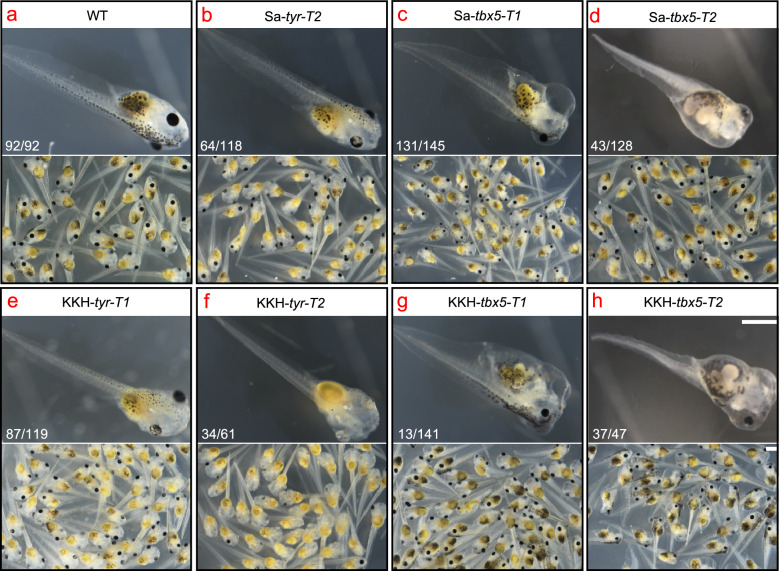
SaCas9 and KKH SaCas9 mediated genome editing are efficient enough for G0 phenotyping in *X. tropicalis*. **a** Wild-type stage 44 tadpoles. **b** Stage 44 tadpoles show minor perturbation of cutaneous and ocular pigmentation upon SaCas9-mediated disruption of *tyr*. **c**, **d** Stage 44 tadpoles show cardiac malformation and edema caused by SaCas9-mediated disruption of *tbx5*. **e**, **f** Stage 44 tadpoles show albinism caused by KKH SaCas9-mediated disruption of *tyr*. **g**, **h** Stage 44 tadpoles show cardiac malformation and edema caused by KKH SaCas9-mediated disruption of *tbx5*. For **b**–**h**, the penetrance of each phenotype is well in line with the corresponding genome editing efficiency. For all panels, the phenotype frequency is given in the representative image. Scale bars, 2 mm. For target site designation, the gene name is in the middle, the prefixes Sa- and KKH- represent SaCas9 and KKH SaCas9, respectively. The suffixes -T1 and -T2 represent the first and the second target site designed in a given gene, respectively

Given the high efficiency of gene disruption induced by SaCas9 and KKH SaCas9, knockout phenotypes could be expected in their mutagenized G0 embryos. We have shown that albinism and heart defects could be easily identified upon direct disruption of *tyr* and *tbx5*, respectively, in *X. tropicalis* embryos [21, 24]. Indeed, varying levels of albinism, heart defects, and heart failure related edema depending on the corresponding gene disruption efficiency (effective frameshifts) were observed in SaCas9- or KKH SaCas9-mediated *tyr* and *tbx5* disruption, respectively (Fig. 5; Additional file 1: Fig. S9). These data indicate that SaCas9 and KKH SaCas9 can be used as tools for G0 phenotyping in *X. tropicalis*.

### SaCas9, KKH SaCas9, and LbCas12/crRNA RNP complexes all have low off-target effects in *X. tropicalis*

To further test the specificity of SaCas9, KKH SaCcas9, and LbCas12/crRNA RNP complexes, we identified genome-wide potential off-target sites with less than five mismatches for 2 SaCas9 targeting sites, 2 KKH SaCas9 targeting sites and 3 LbCas12a targeting sites, with the online software CAS-OFFinder ([27]; Additional file 2: Table S1), of which we selected 23 for T7EI assay (Additional file 2:Table S2). No T7EI signals were detected in these samples (Additional file 2: Table S2, Fig. S10), suggesting low off-target effects of SaCas9, KKH SaCas9, and LbCas12a in *X. tropicalis* embryos, which is consistent with the SpCas9 activity in frog embryos [21].

## Discussion

**Fig. 6:**
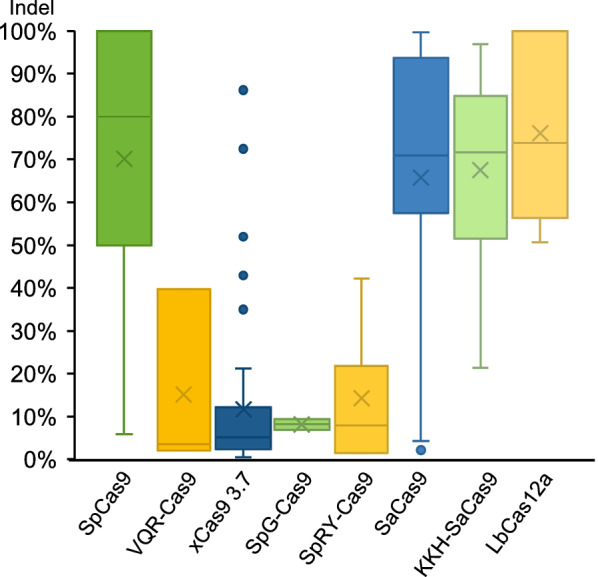
Box plot summary of the targeted genome editing efficiency of 8 Cas variants tested in *X. tropicalis* embryos. The box plot for SpCas9 is based on the data from our previous studies [21, 24]

In this study, we examined mutagenic activity of several natural Cas nucleases as well as engineered Cas9 variants in *X. tropicalis* embryos and found that SaCas9 and KKH SaCas9 are comparable to SpCas9, which could be used as tools even for phenotype assessment in G0 embryos (Figs. 5 and 6). As a known effective tool in frogs, LbCas12a/crRNA RNP complexes are highly effective also for DNA fragment deletion in *X. tropicalis* embryos. We were unable to detect off-target effects for SaCas9, KKH SaCas9, and LbCas12a with T7EI assay, suggesting low off-target effects with these tools in frog embryos. In contrast, all the SpCas9 variants tested showed very limited activity in *X. tropicalis*.

Several factors, such as temperature, chromatin accessibility, and experimental settings, could be related to the low efficacy of VQR Cas9, SpG Cas9, SpRY Cas9, and xCas9 3.7 in *X. tropicalis* embryos. First, *X. tropicalis* embryos are normally incubated at 23 °C, while all those Cas9 variants were evolved and optimized at 37 °C, which may result in reduction of their activity at lower temperatures. It remains to be tested if heat-shock treatment could improve the activity of these variants in *X. tropicalis* embryos. The natural AsCas12a is very ineffective at low temperature and heat-shock treatment can indeed improve the activity of LbCas12a in zebrafish and *X. tropicalis* embryos [14]. Of note, in this study we show that without heat-shock treatment, the LbCas12a/crRNA RNP complexes worked well in *X. tropicalis* embryos at 23 °C for both fragment deletion and indel induction, demonstrating the robustness of this tool for routine applications in frogs. Directed evolution of Cas9 at lower temperature may help identify variants optimized for heterothermic animals, such as frogs, salamanders, and fish. Second, to address if the low efficacy of VQR Cas9, SpG Cas9, SpRY Cas9, and xCas9 3.7 in *X. tropicalis* embryos is corelated with the chromatin microenvironment, more systematic comparison of these variants together with SpCas9, SaCas9, and KKH SaCcas9 for their genome-editing activity within the same genomic region needs to be done. Our data indicate that for the same NGG sites, the targeted genome editing efficiency of xCas9 3.7 is much lower than that of SpCas9 (Additional file 1: Fig. S8, and [24]), suggesting an alternative that these variants in general have evolved an attenuated ability to access target sites in frog chromatin contexts. It has been shown that catalytically dead SpCas9 (SpdCas9) can render the target sites accessible to the other otherwise inactive CRISPR nucleases upon proximal localization to the target site in human cells, which was termed as a proxy-CRISPR strategy [28]. It is yet to be tested if this proxy-CRISPR strategy can improve the genome-editing efficiency of these Cas9 variants in *X. tropicalis* embryos. Third, it should be noted that the doses of gRNAs and mRNAs of these Cas9 variants used in this study were based on our previous optimization for SpCas9 [24], which all did not result in obvious toxicity. In order to be more conclusive, it should be further optimized for each of these Cas9 variants with higher doses and varying ratio of Cas mRNA and gRNA.

## Conclusions

We find that SaCas9 and KKH SaCas9 are robust genome editing tools in *X. tropicalis* embryos. We also show that LbCas12a/crRNA RNP complexes are effective for DNA fragment deletion in frog embryos. These tools expand the CRISPR/Cas genome editing scope in *X. tropicalis* and increase the flexibility for various genome editing applications in *Xenopus.*

## Methods

### Production of Cas9 variants mRNAs, gRNAs, and crRNAs

The human codon-usage optimized VQR-Cas9, SaCas9 and KKH-SaCas9 constructs were purchased from Addgene (Cat. No. 65771, 61591, 70708) and sub-cloned into *pCS2*+ vector. The xCas9 3.7, SpG-Cas9, SpRY-Cas9 and iSpyMac Cas9 were synthesized by IGE Biotechnology (Guangzhou, China) and cloned into *pCS2*+ vector. The detailed sequences of all Cas9 plasmids are shown in Additional file 2: Table S3. All the Cas9 constructs were linearized with NotI and transcribed with the mMessage mMachine SP6 Kit (Thermo Fisher Scientific) to produce capped Cas9 mRNA, which were purified with the RNeasy Mini Kit (Qiagen) according to the RNA clean protocol.

For all the Cas9 sgRNA transcription, the DNA templates were obtained from a pUC57-T7-gRNA scaffold construct by PCR amplification with the overhang forward primer (Sp gRNA: 5′-CTAATACGACTCACTATAGNNNNNNNNNNNNNNNNNNNGTTTTAGAGCTAGAAATAGCAAGTT-3′, Sa gRNA: 5′-CTAATACGACTCACTATAGNNNNNNNNNNNNNNNNNNNNgttttagtactctggaaacagaatc-3′) and universal reverse primer (Sp gRNA: 5′-AAAAAAGCACCGACTCGGTGCCAC-3′, Sa gRNA: 5′-AAAAAATCTCGCCAACAAGTTGAC-3′), transcribed with the Transcript Aid T7 High Yield Transcription Kit (Thermo Fisher Scientific), and purified with the miRNeasy Mini Kit (Qiagen).

LbCas12a protein was purchased from New England Biolabs Inc. LbCas12a crRNA templates were prepared by annealing T7 primer (crRNA-S) with an antisense single strand DNA (crRNA-A) containing a reverse T7 fragment, Cas12a crRNA scaffold, and target sequences, transcribed with the Transcript Aid T7 High Yield Transcription Kit (Thermo Fisher Scientific), and purified with the miRNeasy Mini Kit (Qiagen).

All the target sequences, sequence information for all gRNA and crRNA templates, and PCR primers used for detecting the targeted loci are listed in Additional file 2: Tables S4–S6.

### *Xenopus tropicalis* maintenance, husbandry, and microinjection

*Xenopus tropicalis* frogs were purchased from Nasco (USA) and bred in an in-house facility. Fertilized eggs and embryos were obtained by hormone-induced mating. Cas9 mRNA (300 pg) and sgRNA (50 pg) were co-injected into the animal pole of 1-cell stage embryos in a volume of 2 nl. Cas12a protein (0.75 µg/µl) and crRNA (200 ng/µl) were assembled 10 min in vitro at 37 °C, and were co-injected into the animal pole of 1-cell stage embryos in a volume of 2 nl. During subsequent development, abnormal and dead embryos were sorted out in time.

### T7EI (T7 Endonuclease I) assay, Sanger DNA sequencing, and targeted deep-sequencing

Twenty-four hours after microinjection, we randomly pooled 30 (except otherwise indicated) healthy embryos from each injection, extracted genomic DNA, amplified the on-target or off-target regions by PCR (for off-target regions primers, see Additional file 2: Table S2). Then, the disruption efficiency was detected by T7EI assay, Sanger DNA sequencing, or deep-sequencing.

For T7EI assay, PCR products were denatured and annealed under the following conditions: 95 °C for 5 min, 95–85 °C at − 2 °C/s, 85–25 °C at − 0.1 °C/s, hold at 4 °C. The annealed samples were digested with T7EI (NEB M0302L), separated and measured on an 1.5% TAE agarose gel, and quantified using ImageJ software (NIH).

For Sanger DNA sequencing, the purified PCR products were cloned into the pEASY-Blunt Zero Cloning Kit (TransGen Biotech) by Blunt-ended DNA. Single colonies were randomly picked for Sanger DNA sequencing analyses to detect any indel mutations. The targeted genome editing efficiency was determined by the ratio of mutant to total colonies sequenced.

For deep-sequencing and data analysis, on-target sites were amplified using 2xPhanta Max Master Mix (Vazyme). Wild-type genomic loci were amplified as a control condition. An VAHTS DNA Clean Beads clean-up step (Vazyme) was performed before pooling and 50 ng of DNA from each condition was used for library preparation. Dual-indexed Illumina deep-sequencing libraries were generated using the VAHTS® Universal DNA Library Prep Kit (Vazyme). All deep-sequencing libraries were sequenced on the Illumina HiSeq X10 platform (paired-end 2 × 150-bp reads). The sequencing data were analyzed using CRISPResso2 [29].

### Identification of potential off‑target sites in *X. tropicalis*

All genomic loci containing up to five mismatches compared with the coding sequence for a given gRNA/crRNA followed by the PAMs sequence were identified by mapping the targeted site to *X. tropicalis* genome (JGI 9.0) using Cas-OFFinder [27].

## Supplementary Information

**Additional file 1: Figure S1.** DNA deep-sequencing data show the mutations induced by SaCas9 targeting the genes showed in Fig. 2a. For all the panels, the wild-type sequence is shown at the top with the target site highlighted in yellow and the PAM sequence in blue text. Red dashes indicate deletions and lowercase letters in red indicate insertions or mutations. The numbers in parentheses show the percentage of this sequence in total sequencing reads. For target site designation, the gene name is in the middle, the prefix Sa- represents SaCas9. The suffixes -T1 and -T2 represent the first and the second target site designed in a given gene, respectively. **Figure S2.** DNA deep-sequencing data show the mutations induced by KKH SaCas9 targeting the genes showed in Fig. 2b. For all the panels, the wild-type sequence is shown at the top with the target site highlighted in yellow and the PAM sequence in blue text. Red dashes indicate deletions and lowercase letters in red indicate insertions or mutations. The numbers in parentheses show the percentage of this sequence in total sequencing reads. For target site designation, the gene name is in the middle, the prefix KKH- represents KKH SaCas9. The suffixes -T1 and -T2 represent the first and the second target site designed in a given gene, respectively. **Figure S3.** T7EI assay data show the mutagenic activities of VQR Cas9, SpG Cas9, SpRY Cas9, and iSpyMac in *X. tropicalis* embryos. The targeting sites labelled in red, blue, and green are the effective ones for SpRY Cas9, SpG Cas9, and VQR Cas9, respectively. For target site designation, in addition to the gene name, the name of the Cas9 variant is used as a prefix and in some cases either the PAM motif or T1/T2 is indicated as a suffix. **Figure S4.** DNA deep-sequencing data show the mutations induced by VQR Cas9 targeting the genes showed in Fig. 3a. For all the panels, the wild-type sequence is shown at the top with the target site highlighted in yellow and the PAM sequence in blue text. Red dashes indicate deletions and lowercase letters in red indicate insertions or mutations. The numbers in parentheses show the percentage of this sequence in total sequencing reads. **Figure S5.** DNA deep-sequencing data show the mutations induced by SpG Cas9 targeting the genes showed in Fig. 3a. For all the panels, the wild-type sequence is shown at the top with the target site highlighted in yellow and the PAM sequence in blue text. Red dashes indicate deletions and lowercase letters in red indicate insertions or mutations. The numbers in parentheses show the percentage of this sequence in total sequencing reads. **Figure S6.** DNA deep-sequencing data show the mutations induced by SpRY Cas9 targeting the genes showed in Fig. 3a. For all the panels, the wild-type sequence is shown at the top with the target site highlighted in yellow and the PAM sequence in blue text. Red dashes indicate deletions and lowercase letters in red indicate insertions or mutations. The numbers in parentheses show the percentage of this sequence in total sequencing reads. For target site designation, in addition to the gene name, the name of the Cas9 variant is used as a prefix and the PAM motif is indicated as a suffix. **Figure S7.** T7EI assay data show the mutagenic activities of xCas9 3.7 targeting non-NGG PAM sites in *X. tropicalis* embryos. The targeting sites labelled in red and blue are the effective ones with their effective gray values indicated at the bottom. The blue ones have been further verified by Sanger DNA sequencing. C, control. For target site designation, in addition to the gene name, the name of the Cas9 variant is used as a prefix and the PAM motif is indicated as a suffix. **Figure S8.** NGG xCas9 3.7 is ineffective in *X. tropicalis* embryos. **a** T7EI assay data show the mutagenic activities of xCas9 3.7 targeting NGG PAM sites in *X. tropicalis* embryos. The targeting sites labelled in red have been further verified by Sanger DNA sequencing shown in c. C, control. **b** Sanger DNA sequencing data show the mutations of *bace2* induced by SpCas9. **c** The upper panel shows the Sanger DNA sequencing data of the *bace2* mutation induced by xCas9 3.7 targeting exactly the same site shown in b. The lower panel shows the Sanger DNA sequencing data for xCas9 3.7-*apc*-163 site. For all the panels in b and c, the wild-type sequence is shown at the top with the target site highlighted in yellow and the PAM sequence in blue text. Red dashes indicate deletions and lowercase letters in red indicate insertions or mutations. The numbers in parentheses represent the ratio of this sequence in the total colonies sequenced. **Figure S9.** Sanger DNA sequencing data show the mutations induced by SaCas9 and KKH SaCas9 targeting the genes showed in Fig. 5. For all the panels, the wild-type sequence is shown at the top with the target site highlighted in yellow and the PAM sequence in blue text. Red dashes indicate deletions and lowercase letters in red indicate insertions or mutations. The numbers in parentheses indicate ratio of this sequence in the total colonies sequenced. For target site designation, the gene name is in the middle, the prefixes Sa- and KKH- represent SaCas9 and KKH SaCas9, respectively. The suffixes -T1 and -T2 represent the first and the second target site designed in a given gene, respectively. **Figure S10.** T7EI assay data show no discernible mutagenic activities on potential off-target sites for LbCas12a, SaCas9, and KKH SaCas9 in *X. tropicalis* embryos. Potential off-target (OT) site is indicated as the last suffix. C, control.

**Additional file 2: Table S1.** Computationally identified total number of potential off-target sites in *Xenopus tropicalis* genome with up to 5 mismatches to 7 targets. **Table S2.** 23 potential off-target sites selected for T7EI assay and the corresponding PCR primers for T7EI assay. **Table S3.** Sequences of all Cas9s tested in this study. **Table S4.** List of gRNA targeting sequences for various SpCas9 variants, PCR primers for the amplification of gRNA transcription templates, and PCR primers for detecting the targeted loci. **Table S5.** List of SaCas9 and KKH SaCas9 gRNA targeting sequences, PCR primers for the amplification of gRNA transcription templates, and PCR primers for detecting the targeted loci. **Table S6.** List of LbCas12a crRNA targeting sequences, T7 primer and single-stranded oligodeoxynucleotides used for the generation of crRNA transcription templates, and PCR primers for detecting the targeted loci.

## Data Availability

All deep sequencing data generated in this study have been deposited under accession numbers CRA006908 in the National Genomics Data Center of China.
