# Supplementary material for: Expanding the CRISPR/Cas genome-editing scope in Xenopus tropicalis
Source: Cell Biosci. 2022 Jul 8;12:104. doi: 10.1186/s13578-022-00841-3 (PMC9264700; doi:10.1186/s13578-022-00841-3)
Supplement: Supplementary file 1 — Additional file 1: Figure S1. DNA deep-sequencing data show the mutations induced by SaCas9 targeting the genes showed in Fig. 2a. For all the panels, the wild-type sequence is shown at the top with the target site highlighted in yellow and the PAM sequence in blue text. Red dashes indicate deletions and lowercase letters in red indicate insertions or mutations. The numbers in parentheses show the percentage of this sequence in total sequencing reads. For target site designation, the gene name is in the middle, the prefix Sa- represents SaCas9. The suffixes -T1 and -T2 represent the first and the second target site designed in a given gene, respectively. Figure S2. DNA deep-sequencing data show the mutations induced by KKH SaCas9 targeting the genes showed in Fig. 2b. For all the panels, the wild-type sequence is shown at the top with the target site highlighted in yellow and the PAM sequence in blue text. Red dashes indicate deletions and lowercase letters in red indicate insertions or mutations. The numbers in parentheses show the percentage of this sequence in total sequencing reads. For target site designation, the gene name is in the middle, the prefix KKH- represents KKH SaCas9. The suffixes -T1 and -T2 represent the first and the second target site designed in a given gene, respectively. Figure S3. T7EI assay data show the mutagenic activities of VQR Cas9, SpG Cas9, SpRY Cas9, and iSpyMac in X. tropicalis embryos. The targeting sites labelled in red, blue, and green are the effective ones for SpRY Cas9, SpG Cas9, and VQR Cas9, respectively. For target site designation, in addition to the gene name, the name of the Cas9 variant is used as a prefix and in some cases either the PAM motif or T1/T2 is indicated as a suffix. Figure S4. DNA deep-sequencing data show the mutations induced by VQR Cas9 targeting the genes showed in Fig. 3a. For all the panels, the wild-type sequence is shown at the top with the target site highlighted in yellow and the PAM sequence in [file 13578_2022_841_MOESM1_ESM.pdf]

Sa-*ash1l-T1*

```
GTCCGATGGGACTCGTACAGTTTGTGTGTGGGTTTGTGGCTAGCCGAG WT (25.57)
GTCCGATGGGACTCGT-----TTGTGTGTGGGTTTGTGGCTAGCCGAG -5 (25.54)
GTCCGATGGGACTCGT---------GTGTGGGTTTGTGGCTAGCCGAG -9 (4.64)
GTCCGATGGGACTCGTAC-GTTTGTGTGTGGGTTTGTGGCTAGCCGAG -1 (3.57)
GTCCGATGGGACTCGTACtcGTTTGTGTGTGGGTTTGTGGCTAGCCGAG +1 (3.54)
GTCCGATGGGACTCGT-----------GTGGGTTTGTGGCTAGCCGAG -11 (1.68)
GTCCGATGGGACT---------TGTGTGTGGGTTTGTGGCTAGCCGAG -9 (0.89)
GTCCGATGGG----------------------TTTGTGGCTAGCCGAG -22 (0.87)
```

Sa-*best1-T1*

```
TGGCGTGGCAGCATCTACAAACTGCTGTACAGGGAGTTCCTCATCTTC WT (15.15)
TGGCGTGGCAGCATCTACAAAC--------AGGGAGTTCCTCATCTTC -8 (8.85)
TGGCGTGGCAGCATCTACAAACTGCTG-----GGAGTTCCTCATCTTC -5 (7.16)
TGGCGTGGCAGCATCTACAAACTGC-----AGGGAGTTCCTCATCTTC -5 (5.31)
TGGCGTGGCAGCATCTACAAACTGCTGT---------TCCTCATCTTC -9 (4.70)
TGGCGTGGCAGCATCTACAAACTG--------GGAGTTCCTCATCTTC -8 (3.33)
TGGCGTGGCAGCATCTACAAACTGCTG---AGGGAGTTCCTCATCTTC -3 (1.98)
TGGCGTGGCAGCATCTACAAACTG------AGGGAGTTCCTCATCTTC -6 (1.54)
TGGCGTGGCAGCATCTACA------------GGGAGTTCCTCATCTTC -12(1.36)
TGGCGTGGCAGCATCTACAAACTG----------AGTTCCTCATCTTC -10 (1.15)
```

Sa-*mlh1-T1*

```
AAAGAATCTGATGTGGAAATGGTTGAGGAAGATGAGTCGGTTAAGTCT WT (6.19)
AAAGAATCTGATGTGGAAATGGT---------TGAGTCGGTTAAGTCT -9 (14.85)
AAAGAATCTGATGTGGAAATGGTT------GATGAGTCGGTTAAGTCT -6 (6.25)
AAAGAATCTGATGTGGAAATGGTTGAG----ATGAGTCGGTTAAGTCT -4 (5.96)
AAAGAATCTGATGTGGAAATGGTTGA---AGATGAGTCGGTTAAGTCT -3 (5.00)
AAAGAATCTGATGTGGAAATGGTTGAG---GATGAGTCGGTTAAGTCT -3 (4.79)
AAAGAATCTGATGTGGAAATGG---------ATGAGTCGGTTAAGTCT -9 (3.50)
AAAGAATCTGATGTGGAAATGGTTGAGG------------TTAAGTCT -12 (3.05)
AAAGAATCTGATGTGGAA-------------ATGAGTCGGTTAAGTCT -13 (1.94)
AAAGAATCTGATGTGGAAATGGTTGAG--------GTCGGTTAAGTCT -8 (1.56)
```

Sa-*snf2h-T1*

```
GGAAAATATGACTCACCTTTTGCTTTTCTCTGTAATCTTCACCTTCAA WT (16.69)
GGAAAATATGACTCACCTT--GCTTTTCTCTGTAATCTTCACCTTCAA -2 (45.92)
GGAAAATATGACTCACCTTTT------CTCTGTAATCTTCACCTTCAA -6 (10.43)
GGAAAATATGACTCACCTTT-GCTTTTCTCTGTAATCTTCACCTTCAA -1 (3.66)
GGAAAATATGACTCACCTTTTtGCTTTTCTCTGTAATCTTCACCTTCA +1 (1.33)
GGAAAATATGACT-------TGCTTTTCTCTGTAATCTTCACCTTCAA -6 (1.31)
GGAAAATATGACTCACCT---GCTTTTCTCTGTAATCTTCACCTTCAA -3 (1.18)
```

Sa-*snf2h-T2*

```
GAAAGCTAAGACTCACCATTGCTGTTCTTGATTTTAAGAACCAGTCAA WT (97.05)
GAAAGCTAAGACTCACCAT-GCTGTTCTTGATTTTAAGAACCAGTCAA -1 (0.53)
GAAAGCTAAGACTCAC------TGTTCTTGATTTTAAGAACCAGTCAA -6 (0.31)
```

Sa-*braf-T1*

```
TCCACGTGTAACTCTTCTCCAGTGATCCAGCTTATATCTGTATCCCAG WT (5.99)
TCCACGTGTAACTCTTC---AGTGATCCAGCTTATATCTGTATCCCAG -3 (12.08)
TCCACGTGTAACTCTTCTC-AGTGATCCAGCTTATATCTGTATCCCAG -1 (10.47)
TCCACGTGTAACTCTTCTCCAG--------CTTATATCTGTATCCCAG -8 (8.28)
TCCACGTG----------------ATCCAGCTTATATCTGTATCCCAG -16 (5.02)
TCCACGTGTAACTCTTC----------CAGCTTATATCTGTATCCCAG -10 (3.88)
TCCACGTGTAACTCTTCTCttCAGTGATCCAGCTTATATCTGTATCCC +2 (2.83)
TCCACGTGTAACTCTTC-----------AGCTTATATCTGTATCCCAG -11 (2.49)
TCCACGTGTAAC---------GTGATCCAGCTTATATCTGTATCCCAG -9 (1.59)
```

Sa-*gdf5-T1*

```
CCCCTTAATTACTCCTCATGAATACATGTTATCACTGTACCGGACTCT WT (5.83)
CCCCTTAATTACTCCTCAT----ACATGTTATCACTGTACCGGACTCT -4 (18.98)
CCCCTTAATTACTCCTCATG--------TTATCACTGTACCGGACTCT -8 (10.33)
CCCCTTAATTAC-------------ATGTTATCACTGTACCGGACTCT -13 (7.12)
CCCCTTAATTACTCCTCAT--ATACATGTTATCACTGTACCGGACTCT -2 (2.43)
CCCCTTAATTACTCCTCA---ATACATGTTATCACTGTACCGGACTCT -3 (1.06)
CCCCTTAATTACTCCT-------ACATGTTATCACTGTACCGGACTCT -7 (1.04)
```

Sa-*hdac4-T1*

```
GAGGGTGATGTGTATAGAGGGAGCTGCGATACTGAGTTCTCCCTGTTC WT (0)
GAGGGTGATGTGTATAGAGGGAGCTG--------AGTTCTCCCTGTTC -8 (21.53)
GAGGGTGATGTGTATAGAGGGAGCTGCG--------TTCTCCCTGTTC -8 (10.72)
GAGGGTGATGTGTATAGAGGGAGCT-----ACTGAGTTCTCCCTGTTC -5 (5.17)
GAGGGTGATGTGTATAGAGGGA-------TACTGAGTTCTCCCTGTTC -7 (4.94)
GAGGGTGATGTGTATAGAGGGAGCTGC-----TGAGTTCTCCCTGTTC -5 (4.71)
GAGGGTGATGTGTATAGAGGGAG-------------TTCTCCCTGTTC -13 (4.12)
GAGGGTGATGTGTATAGAGGGAGCTGCG-TTCTGAGTTCTCCCTGTTC -1 (2.76)
GAGGGTGATGTGTATAGAG-----------------TTCTCCCTGTTC -17 (1.82)
GAGGGTGATGTGTATAGAGGGAGCTGCGggATACTGAGTTCTCCCTGT +2 (1.52)
GAGGGTGATGTGTATAGAGGGAGCTGCGA------GTTCTCCCTGTTC -6 (1.29)
GAGGGTGATGTGTATAGAGGGAGCTGC--TACTGAGTTCTCCCTGTTC -2 (1.22)
GAGGGTGATGTGTATAGAGGGAGCT-------------CTCCCTGTTC -13 (1.06)
```

Sa-*mlh1-T2*

```
AAGAAGAAGAATTATAAATTTGACAAGTATACTGAGTCTGCAGAAGAA WT (95.35)
AAGAAGAAGAATTATAAATTTGACAAG--TACTGAGTCTGCAGAAGAA -2 (0.58)
AAGAAGAAGAATTATAAATTTGACA---------AGTCTGCAGAAGAA -9 (0.53)
AAGAAGAAGAATTATAAATTTGACAA---TACTGAGTCTGCAGAAGAA -3 (0.45)
AAGAAGAAGAATTATAAATTTG--------ACTGAGTCTGCAGAAGAA -8 (0.36)
AAGAAGAAGAATTATAAATTTGACAAGT---CTGAGTCTGCAGAAGAA -3 (0.22)
AAGAAGAAGAATTATAAATT------------TGAGTCTGCAGAAGAA -12 (0.16)
AAGAAGAAGAATTATAAATTTGACA----TACTGAGTCTGCAGAAGAA -4 (0.15)
AAGAAGAAGAATTATAAATTTGACAA--ATACTGAGTCTGCAGAAGAA -2 (0.15)
```

Sa-*musk-T1*

```
TGAGAGAATTACTCCATCTCTGATATTTTCATCAACCAATTCAGAGAG WT (0)
TGAGAGAATTACTCCATCT--GATATTTTCATCAACCAATTCAGAGAG -2 (53.89)
TGAGAGAATTACT--------GATATTTTCATCAACCAATTCAGAGAG -8 (2.84)
TGAGAGAATTACTCCATC---------------AACCAATTCAGAGAG -15 (2.67)
TGAGAGAATTACTCCAT-------ATTTTCATCAACCAATTCAGAGAG -7 (1.81)
TGAGAGAATTACTCCAT----GATATTTTCATCAACCAATTCAGAGAG -4 (1.67)
TGAGAGAATTACTCCAT---ccATATTTTCATCAACCAATTCAGAGAG -3 (1.43)
TGAGAGAATTACTCC-----TGATATTTTCATCAACCAATTCAGAGAG -5 (1.12)
```

Fig. S1. DNA deep-sequencing data show the mutations induced by SaCas9 targeting the genes showed in Fig. 2a. For all the panels, the wild-type sequence is shown at the top with the target site highlighted in yellow and the PAM sequence in blue text. Red dashes indicate deletions and lowercase letters in red indicate insertions or mutations. The numbers in parentheses show the percentage of this sequence in total sequencing reads. For target site designation, the gene name is in the middle, the prefix Sa- represents SaCas9. The suffixes -T1 and -T2 represent the first and the second target site designed in a given gene, respectively.

KKH-*ash1l-T1*

```
GAAAGTGGCGGCGAGGCTGGAGACAGAACCTTGCAGTCTGGGCTGGAC WT (3.83)
GAAAGTGGCGGCGAGGCTGGAGACA----------GTCTGGGCTGGAC -10 (22.82)
GAAAGTGGCGGCGAGGCTGG---------------------GCTGGAC -21 (3.46)
GAAAGTGGCGGCGAGGCTGGAGACAGAA-------GTCTGGGCTGGAC -7 (3.30)
GAAAGTGGCGGCGAGGCTGGA--------------------------C -26 (2.61)
GAAAGTGGCGGCGAGGCTGGAGACAG-------CAGTCTGGGCTGGAC -7 (2.42)
GAAAGTGGCGGCGAGGCTGGAGACAGA-CCTTGCAGTCTGGGCTGGAC -1 (1.95)
GAAAGTGGCGGCGAGGCTGGAG--------------------CTGGAC -20 (1.83)
GAAAGTGGCGGCGAGGCTGG--------------AGTCTGGGCTGGAC -14 (1.73)
GAAAGTGGCGGCGAGGCTGGAGACAGA--CTTGCAGTCTGGGCTGGAC -2 (1.69)
GAAAGTGGC-------------------------AGTCTGGGCTGGAC -25 (1.68)
GAAAGTGGCGGCGAGGCTGGAGAC------TTGCAGTCTGGGCTGGAC -6 (1.48)
```

KKH-*ash1l-T2*

```
CATGGTGCATGTAGGAGGGAGGGGGCAGCGGGGTAGTAAATGAGGGGG WT (38.52)
CATGGTGCATGTAGGAGGGAGG---------GGTAGTAAATGAGGGGG -9 (8.10)
CATGGTGCATGTAGGAGGGAGGGGGC--------AGTAAATGAGGGGG -8 (4.43)
CATGGTGCATGTAGGAGGGAGGGG-------GGTAGTAAATGAGGGGG -7 (4.38)
CATGGTGCATGTAGGAGGGAGGGGG----------------------- -23 (3.98)
CATGGTGCATGTAGGAGGG-------------GTAGTAAATGAGGGGG -13 (3.63)
CATGGTGCATGTAGG----------------GGTAGTAAATGAGGGGG -16 (3.00)
CATGGTGCATGTAGGAGGGAGGGGG--------TAGTAAATGAGGGGG -8 (2.27)
CATGGTGCATGTAGGAGGGAG------------TAGTAAATGAGGGGG -12 (2.11)
CATGGTGCATGTAGGAGGGAGG-----------TAGTAAATGAGGGGG -11 (1.66)
CATGGTGCATGTAGGGGGAAGGGGGC------------GGAGGATAGA -12 (1.61)
CATGGTGCATG--------------------GGTAGTAAATGAGGGGG -20 (1.58)
```

KKH-*best1-T1*

```
TACTGTGATAACTATGCAGAGCTGATTCCTGTGTCATTTGTATTAGGT WT (59.79)
TACTGTGATAACTATGCAG--CTGATTCCTGTGTCATTTGTATTAGGT -2 (17.07)
TACTGTGATAACTATGC-----TGATTCCTGTGTCATTTGTATTAGGT -5 (6.89)
TACTGTGATAACTATGCAGA-----TTCCTGTGTCATTTGTATTAGGT -5 (2.08)
```

KKH-*hdac4-T1*

```
CCACAGATGCACTGGTGTTTTAACATCAATGTATCATACACAAGGCCT WT (34.65)
CCACAGATGCACTGGTGTT--AACATCAATGTATCATACACAAGGCCT -2 (34.07)
CCACAGATGCAC------------ATCAATGTATCATACACAAGGCCT -12 (1.97)
CCACAGATGCACTGGTGT--------------ATCATACACAAGGCCT -14 (1.46)
CCACAGATGCACTGGT----TAACATCAATGTATCATACACAAGGCCT -4 (1.03)
```

KKH-*musk-T1*

```
TACAAGCAAAACTTGGCCTATCAGATGGCATCTTGCTCCAGCAGAGTC WT (5.69)
TACAAGCAAAACTTGGCC---------------------AGCAGAGTC -21 (34.15)
TACAAGCAAAACTTGGCC------------TCTTGCTCCAGCAGAGTC -12 (28.46)
TACAAGCAAAACTTGGC------------ATCTTGCTCCAGCAGAGTC -12 (13.82)
TACAAGCAAAACTTGGCCT--CAGATGGCATCTTGCTCCAGCAGAGTC -2 (5.69)
TACAAGCAAAAC----------AGATGGCATCTTGCTCCAGCAGAGTC -10 (1.63)
TACAAGCAAAACTTGacagtaaaATCAGATGGCATCTTGCTCCAGCAG +4 (1.63)
```

KKH-*braf-T1*

```
CGCTGGTGGAACTTGTACCCGCACGTCTGGCAGCGGAAGCCCTGGAAC WT (15.27)
CGCTGGTGGAACTTGTAC------GTCTGGCAGCGGAAGCCCTGGAAC -6 (24.69)
CGCTGGTGGAACTTGTACC-GCACGTCTGGCAGCGGAAGCCCTGGAAC -1 (5.51)
CGCTGGTGGAACTTGTACC---ACGTCTGGCAGCGGAAGCCCTGGAAC -3 (4.16)
CGCTGGTGGAACTTGTAC--GCACGTCTGGCAGCGGAAGCCCTGGAAC -2 (4.09)
CGCTGGTGGAACTTGTACC-----GTCTGGCAGCGGAAGCCCTGGAAC -5 (2.44)
CGCTGGTGGAACTTGT----------CTGGCAGCGGAAGCCCTGGAAC -10 (2.04)
CGCTGGTGGAACT------CGCACGTCTGGCAGCGGAAGCCCTGGAAC -6 (1.56)
CGCTGGTGGAACTTGTACCaCGCACGTCTGGCAGCGGAAGCCCTGGAA +1 (1.27)
```

KKH-*braf-T2*

```
GGTAACGCACACTTACCTGAGGACTCCTACTTCATTTTTAAAGGCCTG WT (75.44)
GGTAACGCACACT------------CCTACTTCATTTTTAAAGGCCTG -12 (4.90)
GGTAACGCACACTTACCTG--GACTCCTACTTCATTTTTAAAGGCCTG -2 (3.21)
GGTAACGCACACTTACCT----------ACTTCATTTTTAAAGGCCTG -10 (1.28)
GGTAACGCACACTTACC--AGGACTCCTACTTCATTTTTAAAGGCCTG -2 (0.98)
```

KKH-*nfyb-T1*

```
GAAATCCCTAACTGAGACGTATCTGTTGTAGAGCTATCCCCGTCCATC WT (40.14)
GAAATCCCTAACTG-----------TTGTAGAGCTATCCCCGTCCATC -11 (11.18)
GAAATCCCTA-----------TCTGTTGTAGAGCTATCCCCGTCCATC -11 (9.76)
GAAATCCCTAACTGAGA---------------GCTATCCCCGTCCATC -15 (2.20)
GAAATCCCTAACTGAGACGT------TGTAGAGCTATCCCCGTCCATC -6 (1.59)
GAAATCCCTAACTGAGAC-----TGTTGTAGAGCTATCCCCGTCCATC -5 (1.58)
GAAATCCCTAACT-------ATCTGTTGTAGAGCTATCCCCGTCCATC -7 (1.15)
GAAATCCCTAA----------TCTGTTGTAGAGCTATCCCCGTCCATC -10 (1.04)
```

KKH-*snf2h-T1*

```
TTCCTCTTTAACTGCGTCTCCGCCCGGCTCTTGTTTCGCCTGATCGCC WT (14.71)
TTCCTCTTTAACTGC--------CCGGCTCTTGTTTCGCCTGATCGCC -8 (10.80)
TTCCTCTTTAACTGCG------CCCGGCTCTTGTTTCGCCTGATCGCC -6 (9.93)
TTCCTCTTTAACTGCGTC--CGCCCGGCTCTTGTTTCGCCTGATCGCC -2 (6.80)
TTCCTCTTTAACTGCGTC-----CCGGCTCTTGTTTCGCCTGATCGCC -5 (2.66)
TTCCTCTTTAACTGCGTCT------------TGTTTCGCCTGATCGCC -12 (2.33)
TTCCTCTTTAACTGCG----------GCTCTTGTTTCGCCTGATCGCC -10 (2.21)
TTCCTCTTTAACTGCGTC------CGGCTCTTGTTTCGCCTGATCGCC -6 (1.98)
TTCCTCTTTAACTGCGTCTC-GCCCGGCTCTTGTTTCGCCTGATCGCC -1 (1.93)
```

KKH-*snf2h-T2*

```
TTCTAAGGGAACTTTCACCCATGTTTGAGAGTTTCTCATTCATTTCTG WT (3.02)
TTCTAAGGGAACTTTCA----TGTTTGAGAGTTTCTCATTCATTTCTG -4 (20.22)
TTCTAAGGGAACTTTCACC-ATGTTTGAGAGTTTCTCATTCATTTCTG -1 (4.93)
TTCTAAGGGAACTTTCAC--ATGTTTGAGAGTTTCTCATTCATTTCTG -2 (4.16)
TTCTAAGGGAACTTT-----------GAGAGTTTCTCATTCATTTCTG -11 (3.83)
TTCTAAGGGAACTTTCAC-gtTGTTTGAGAGTTTCTCATTCATTTCTG -1 (3.08)
TTCTAAGGGA--------------------GTTTCTCATTCATTTCTG -20 (2.67)
TTCTAAGGGAACTTTt---CATGTTTGAGAGTTTCTCATTCATTTCTG -3 (2.24)
TTCTAAGGGAACTTTCACtttCATGTTTGAGAGTTTCTCATTCATTTC +2 (2.20)
TTCTAAGGGAACTTTC-------------------TCATTCATTTCTG -19 (2.21)
```

Fig. S2. DNA deep-sequencing data show the mutations induced by KKH SaCas9 targeting the genes showed in Fig. 2b. For all the panels, the wild-type sequence is shown at the top with the target site highlighted in yellow and the PAM sequence in blue text. Red dashes indicate deletions and lowercase letters in red indicate insertions or mutations. The numbers in parentheses show the percentage of this sequence in total sequencing reads. For target site designation, the gene name is in the middle, the prefix KKH- represents KKH SaCas9. The suffixes -T1 and -T2 represent the first and the second target site designed in a given gene, respectively.

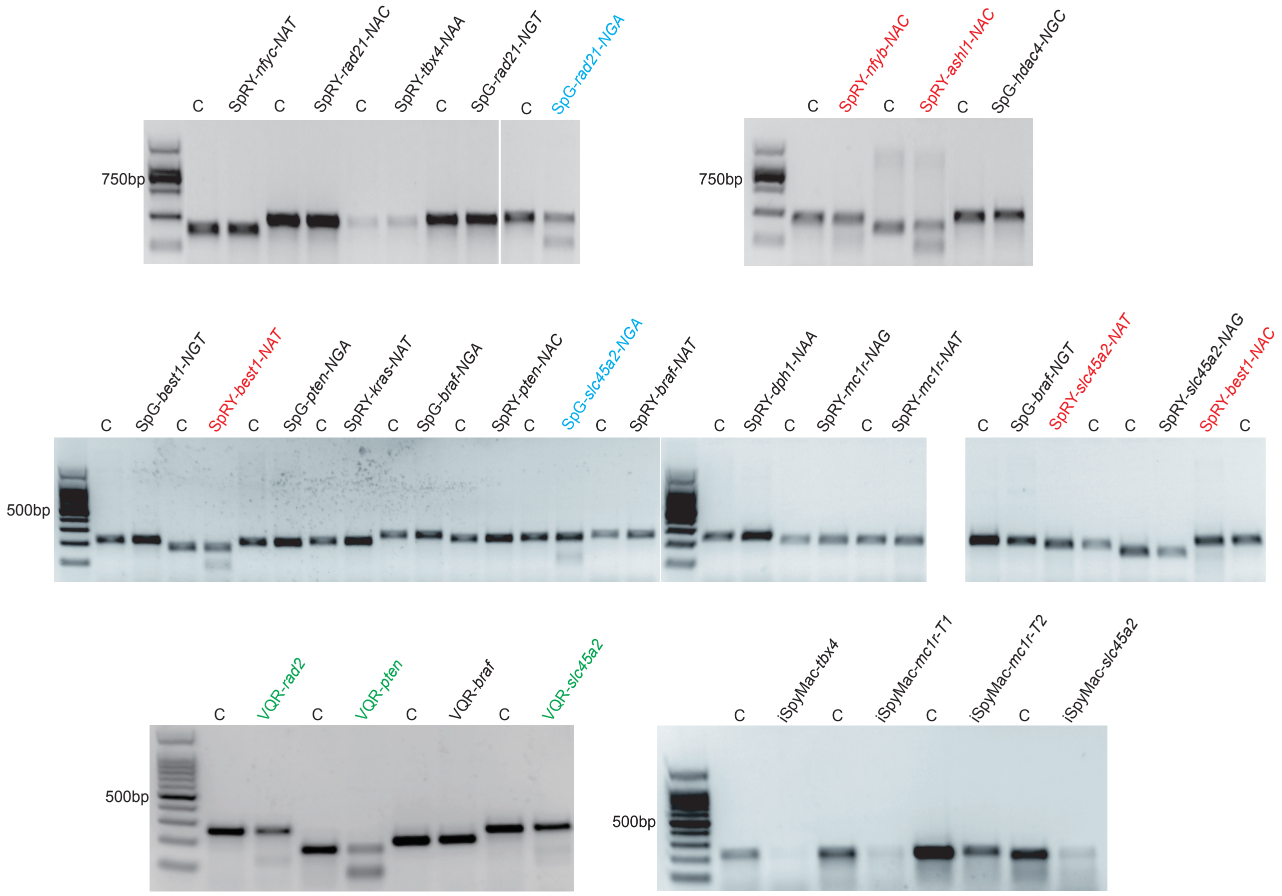

Fig. S3. T7EI assay data show the mutagenic activities of VQR Cas9, SpG Cas9, SpRY Cas9, and iSpyMac in *X. tropicalis* embryos. The targeting sites labelled in red, blue, and green are the effective ones for SpRY Cas9, SpG Cas9, and VQR Cas9, respectively. For target site designation, in addition to the gene name, the name of the Cas9 variant is used as a prefix and in some cases either the PAM motif or T1/T2 is indicated as a suffix.

VQR-*pten*

```
CCTTCAAGCCTCTCTGCAGGAAAGCCCATGGCAATGATGTTTGG WT (59.57)
CCTTCAAGCCTCTC---AGGAAAGCCCATGGCAATGATGTTTGG -3 (11.72)
CCTTCAAGCCTCTCTG---GAAAGCCCATGGCAATGATGTTTGG -3 (8.93)
CCTTC----------------AAGCCCATGGCAATGATGTTTGG -16(2.50)
CCTTCAAGCCTCTCTC-AGGAAAGCCCATGGCAATGATGTTTGG -1 (2.39)
CCTTCAAGCCTCTCTG--GAAAAGCCCATGGCAATGATGTTTGG -2 (0.89)
CCTTCAAGCCTC-----AGGAAAGCCCATGGCAATGATGTTTGG -5 (0.67)
CCTTCAAGCCTCTCTctCAGGAAAGCCCATGGCAATGATGTTTG +1 (0.57)
```

VQR-*rad21*

```
GGTCAAAGTCATGGAACTCCTCAGGCAGAGTGATGGCATTGTAT WT (96.79)
GGTCAAAGTCATGGAACTCCTCAGGC-----------ATTGTAT -11 (0.40)
GGTCAAAGTCATGGAACTCCTCAGG---AGTGATGGCATTGTAT -3 (0.22)
GGTCAAAGTCATGGAACTCCTCAG------TGATGGCATTGTAT -6 (0.21)
GGTCAAAGTCATGGAACTCCTCAGGCAaGAGTGATGGCATTGTA +1 (0.16)
GGTCAAAGTCATGGAACTCCTCAG----AGTGATGGCATTGTAT -4 (0.15)
GGTCAAAGTCATGGAACTCCTCAGGC---GTGATGGCATTGTAT -3 (0.12)
```

VQR-*slc45a2*

```
CCATGCCTGCTCATTATCGCTACCTGTGCCTGAGCCACTTAATT WT (95.18)
CCATGCCTGCTCATTATCGCTACC------TGAGCCACTTAATT -6 (1.70)
CCATGCCTGCTCATTATCGCTACCTGTtGCCTGAGCCACTTAAT +1 (0.29)
CCATGCCTGCTCATTATCGCTACCTG--CCTGAGCCACTTAATT -2 (0.25)
CCATGCCTGCTCATTATCGCT------GCCTGAGCCACTTAATT -6 (0.24)
CCATGCCTGCTCATTATCG---------CCTGAGCCACTTAATT -9 (0.23)
CCATGCCTGCTCATTATCGCTACCTGaGCCTGAGCCACTTAATT 0 (0.21)
CCATGCCTGCTCATTATCGCTACCTGG-CCTGAGCCACTTAATT -1 (0.14)
```

Fig. S4. DNA deep-sequencing data show the mutations induced by VQR Cas9 targeting the genes showed in Fig. 3a. For all the panels, the wild-type sequence is shown at the top with the target site highlighted in yellow and the PAM sequence in blue text. Red dashes indicate deletions and lowercase letters in red indicate insertions or mutations. The numbers in parentheses show the percentage of this sequence in total sequencing reads.

SpG-*slc45a2-NGA*

```
TACCATGCCTGCTCATTATCGCTACCTGTGCCTGAGCCACTTAATTGG WT (91.62)
TACCATGCCTGCTCATTATCGCTA------CCTGAGCCACTTAATTGG -6 (2.57)
TACCATGCCTGCTCATTATCGCTACCTGTtGCCTGAGCCACTTAATTG +1 (0.90)
TACCATGCCTGCTCATTATCGC---------CTGAGCCACTTAATTGG -9 (0.70)
TACCATGCCTGCTCATTATCGCTACCT--GCCTGAGCCACTTAATTGG -2 (0.56)
TACCATGCCTGCTCATTATCGCT------GCCTGAGCCACTTAATTGG -6 (0.55)
TACCATGCCTGCTCATTATCGCTACCTGaGCCTGAGCCACTTAATTGG M1 (0.38)
```

SpG-*rad21-NGA*

```
CTGGTCAAAGTCATGGAACTCCTCAGGCAGAGTGATGGCATTGTATGC WT (89.35)
CTGGTCAAAGTCATGGAACTCCTCAG-----------GCATTGTATGC -11 (1.35)
CTGGTCAAAGTCATGGAACTCCTCAG------TGATGGCATTGTATGC -6 (0.86)
CTGGTCAAAGTCATGGAACTCCTCAGGCAaGAGTGATGGCATTGTATG +1 (0.76)
CTGGTCAAAGTCATGGAACTCCTCAG----AGTGATGGCATTGTATGC -4 (0.74)
CTGGTCAAAGTCATGGAACTCCTCAGG---AGTGATGGCATTGTATGC -3 (0.63)
CTGGTCAAAGTCATGGAACTCCTCAG-----GTGATGGCATTGTATGC -5 (0.32)
CTGGTCAAAGTCATGGAACTCCTCAGGC---GTGATGGCATTGTATGC -2 (0.30)
```

Fig. S5. DNA deep-sequencing data show the mutations induced by SpG Cas9 targeting the genes showed in Fig. 3a. For all the panels, the wild-type sequence is shown at the top with the target site highlighted in yellow and the PAM sequence in blue text. Red dashes indicate deletions and lowercase letters in red indicate insertions or mutations. The numbers in parentheses show the percentage of this sequence in total sequencing reads.

SpRY-*ash1l-NAC*

```
GCTCTCCTGCTCAGGCAGCTGGCCCAGCTTGGCACACTGACCGGTGCT WT (79.51)
GCTCTCCTGCTCAGGCAGC----------TGGCACACTGACCGGTGCT -10 (3.88)
GCTCTCCTGCTCAGGCAGCTGGCCCAGCT-GGCACACTGACCGGTGCT -1 (2.05)
GCTCTCCTGCTCAGGCAGCTGGCCCA-----GCACACTGACCGGTGCT -5 (1.90)
GCTCTCCTGCTCAGGCAGCTGGCC----------CACTGACCGGTGCT -10 (1.28)
GCTCTCCTGCTCAGGCAGCTGGCCCAG---------CTGACCGGTGCT -9 (0.97)
GCTCTCCTGCTCAGGCAGCTGGCCCAGCTTtGGCACACTGACCGGTGC +1 (0.74)
GCTCTCCTGCTCA-----------------GGCACACTGACCGGTGCT -17 (0.54)
```

SpRY-*nfyb-NAC*

```
GACGTATCTGTTGTAGAGCTATCCCCGTCCATCTAAAAGAAACAAACA WT (89.35)
GACGTATCTGTTGTA------TCCCCGTCCATCTAAAAGAAACAAACA -6 (1.66)
GACGTATCTGTTGT--------------CCATCTAAAAGAAACAAACA -14 (0.69)
GACGTATCTGT-----------------CCATCTAAAAGAAACAAACA -17 (0.54)
GACGTATCCCC---------------GTCCATCTAAAAGAAACAAACA -15 (0.50)
GACGTATCTATC-----------CCCGTCCATCTAAAAGAAACAAACA -11 (0.29)
GACGTATCTGTTGTAG----ATCCCCGTCCATCTAAAAGAAACAAACA -4 (0.24)
GACGTATCTGTTGTAGAGCcTATCCCCGTCCATCTAAAAGAAACAAAC +1 (0.22)
```

SpRY-*best1-NAC*

```
TCTTCTTTTTGATAGGAGCTAAATTCTCTGAGGACACAATGTGGCAGG WT (93.20)
TCTTCTTTTTGATAGGAGCTAAATTCTCctaccaagctggcccagctt +27 (0.49)
TCTTCTTTTTGATAGGAGCTAAA----------------TGTGGCAGG -16 (0.48)
TCTTCTTTTTGAT-----------------AGGACACAATGTGGCAGG -17 (0.32)
TCTTCTTTTTGATAGGAGCTAAATTCCCG-AGGACACAATGTGGCAGG -1 (0.31)
TCTTCTTTTTGATAGGAGCTAAATTCTCTtGAGGACACAATGTGGCAG +1 (0.28)
TCTTCTTTTTGATAGGAGCTAAATTatagGAGGACACAATGTGGCAGG M4 (0.26)
TCTTCTTTTTGATAGGAGCTAA-----------------TGTGGCAGG -17 (0.22)
```

SpRY-*slc45a2-NAT*

```
CCTATGCAGAACATAACTCCACAGCATATCTAACCTATGAAAGAGGAG WT (98.96)
CCTATGCAGAACATAAC-----AGCATATCTAACCTATGAAAGAGGAG -5 (0.13)
CCTATGCAGAACATAACata-----ATATCTAACCTATGAAAGAGGAG -5 (0.1)
CCTATGCAGAACATAAC--CACAGCATATCTAACCTATGAAAGAGGAG -2 (0.08)
```

Fig. S6. DNA deep-sequencing data show the mutations induced by SpRY Cas9 targeting the genes showed in Fig. 3a. For all the panels, the wild-type sequence is shown at the top with the target site highlighted in yellow and the PAM sequence in blue text. Red dashes indicate deletions and lowercase letters in red indicate insertions or mutations. The numbers in parentheses show the percentage of this sequence in total sequencing reads. For target site designation, in addition to the gene name, the name of the Cas9 variant is used as a prefix and the PAM motif is indicated as a suffix.

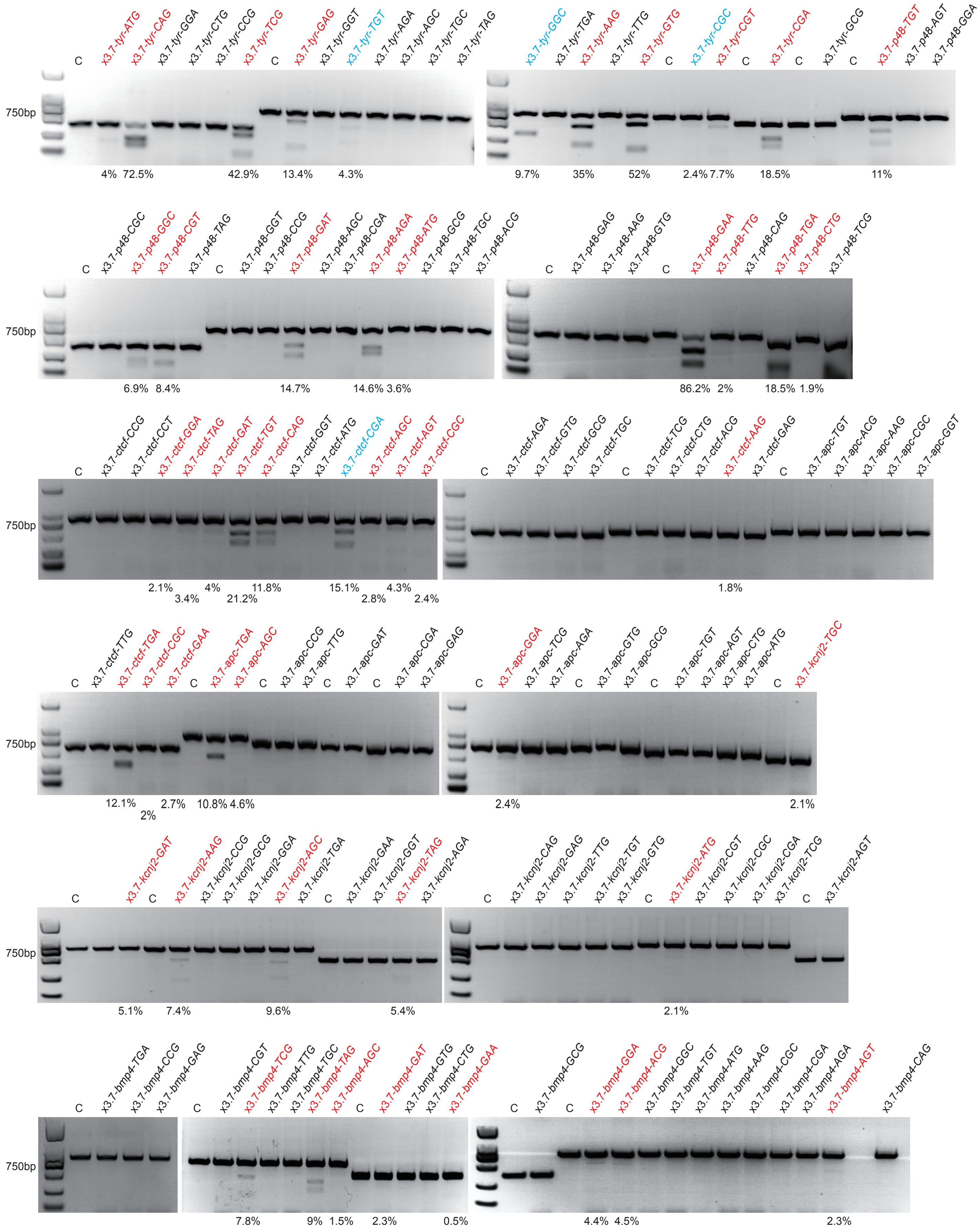

Fig. S7. T7EI assay data show the mutagenic activities of xCas9 3.7 targeting non-NGG PAM sites in *X. tropicalis* embryos. The targeting sites labelled in red and blue are the effective ones with their effective gray values indicated at the bottom. The blue ones have been further verified by Sanger DNA sequencing. C, control. For target site designation, in addition to the gene name, the name of the Cas9 variant is used as a prefix and the PAM motif is indicated as a suffix.

a

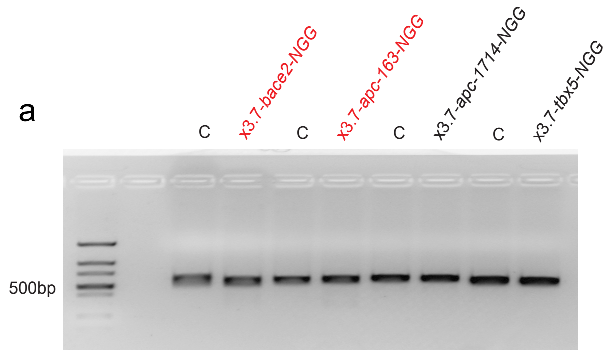

b

SpCas9-*bace2* (16/20)

```
GTTGAATATTCTTCTTCTAGATGAACATCTTGGTGGACACGGGAAGCAGTAATTTCGCTG wt (4/20)
GTTGAATATTCTTCTTCTAGATGAACATCTTGGTGG-----GGAAGCAGTAATTTCGCTG -5 (2/20)
GTTGAATATTCTTCTTCTAGATGAACATCTTGGT-------GGAAGCAGTAATTTCGCTG -7 (2/20)
GTTGAATATTCTTCTTCTAGATGAACATCTTGG---------GAAGCAGTAATTTCGCTG -9 (2/20)
GTTGAATATTCTTCTTCTAGATGAACATCTTGGTGG------GAAGCAGTAATTTCGCTG -6 (2/20)
GTTGAATATTCTTCTTCTAGATGAACATCTagaagaa---GGGAAGCAGTAATTTCGCTG -3 (1/20)
GTTGAATATTTTTCTTCTAGATGAACATCTTGGTGG-----GtAAGCAGTAATTTCGCTG -5 (1/20)
GTTGAATATTCTTCTTTTAGATGAACAT-atagaagaagCGGGAAGCAGTAATTTCGCTG -1 (1/20)
GTTGAATATTCTTCTTCTAGATGAACATCTTGGTGG----GGGAAGCAGTAATTTCGCTG -4 (1/20)
GTTGAATATTCTTCTTCTAGATGAACATCTTGG------------------------CTG -24 (1/20)
GTTGAATATTCTTCTTCTAGATGAACATCTTGGTGatgaACACGGGAAGCAGTAATTTCG +3 (1/20)
GTTGAATATTCTTCTTCTAGATGAACATCTagatgaacaACACGGGAAGCAGTAATTTCG +3 (1/20)
GTTGAATATTCTTCTTCTAGATGAACATCTTGGTGGgtgtaACACGGGAAGCATTAATTT +5 (1/20)
```

c

xCas9 3.7-*bace2* (1/8)

```
GTTGAATATTCTTCTTCTAGATGAACATCTTGGTGGACACGGGAAGCAGTAATTTCGCTG wt (7/8)
GTTGAATATTCTTCTTCTAGATGAACATCTTGGT-------GGAAGCAGTAATTTCGCTG -7 (1/8)
```

xCas9 3.7-*apc-163* (1/20)

```
CAAAGAAGAGGCCTTTTAAATGGAAGCAGAGAAAGTACTGGGTATCTGGAAGAACTTGAG wt (19/20)
CAAAGAAGAGGCCTTTTAAATGGAAGCAGA-----------------------ACTTGAG -23 (1/20)
```

Fig. S8. NGG xCas9 3.7 is ineffective in *X. tropicalis* embryos. **(a)** T7EI assay data show the mutagenic activities of xCas9 3.7 targeting NGG PAM sites in *X. tropicalis* embryos. The targeting sites labelled in red have been further verified by Sanger DNA sequencing shown in c. C, control. **(b)** Sanger DNA sequencing data show the mutations of *bace2* induced by SpCas9. **(c)** The upper panel shows the Sanger DNA sequencing data of the *bace2* mutation induced by xCas9 3.7 targeting exactly the same site shown in b. The lower panel shows the Sanger DNA sequencing data for xCas9 3.7-*apc*-163 site. For all the panels in b and c, the wild-type sequence is shown at the top with the target site highlighted in yellow and the PAM sequence in blue text. Red dashes indicate deletions and lowercase letters in red indicate insertions or mutations. The numbers in parentheses represent the ratio of this sequence in the total colonies sequenced.

Sa-*tyr-T2* (8/19)

```
GAAACTCACAGGAGATGAGAATTTTACCATCCC WT (11/19)
GAAACTCACAG---ATGAGAATTTTACCATCCC -3 (3/19)
GAAACTCACAcagaattctcatctcctgtttta -34(2/19)
GAAACTCACAGG----GAGAATTTTACCATCCC -4 (1/19)
GAAACTCACAG---ATGAGAATTTTACCATCCC -3 (1/19)
GAAACTCACAGGA---GAGAATTTTACCATCCC -3 (1/19)
GAAACTCACAGatGAGATGAGAATTTTACCATC +2 (1/19)
```

Sa-*tbx5-T1* (14/15)

```
CAGACTCACCAGCCACAGGCGCACACTGGATGA WT (1/15)
CAGACTCACCA----CAGGCGCACACTGGATGA -4 (5/15)
CAGACTCACCAG------GCGCACACTGGATGA -6 (3/15)
CAGACTCACCAGgCCACAGGCGCACACTGGATG +1 (1/15)
CAGACTCAC-----------------TGGATGA -17(1/15)
CAGACTCACCAGACACcacAGGCGCACACTGGA +3 (1/15)
ggtgaactggcaCCACAGGCGCACACTGGATGA -29(1/15)
ttgtatgc--------AGGCGCACACTGGATGA -8 (1/15)
CAGACTCACCA--------------CTGGATGA -13(1/15)
```

Sa-*tbx5-T2* (13/14)

```
CCAATGCATATCCTTCCCTGCCCCATGAGTCTG WT (1/14)
CCAATGCATATCCTTCCC------ATGAGTCTG -6 (3/14)
G--------------------------------  -150(1/14)
CCAATGCATATCatgag----------------  -33(1/14)
CCAATGCATATCCTTCCCTGC------------  -29(1/14)
CCAATGCATATCCTTC-------------TCTG -13(1/14)
CCAATGCATATCCTTCC-------ATGAGTCTG -7 (1/14)
CCAATGCATATCCTTCCC-----CATGAGTCTG -5 (1/14)
CCAATGCATATCCTTCCCT--CCCATGAGTCTG -2 (1/14)
CCAATGCATATCCTTCCCTtCCCCATGAGTCTG M1 (1/14)
CCAATGCATATCCTTCCCTGCgagtgcattgga +24(1/14)
CCAATGCATATCCTTCCCTGCacatatccttcc +15(1/14)
```

KKH-*tyr-T1* (8/13)

```
TCCTGACCAGCTCTGCTACTGGCCCTCAGTTTC  WT (5/13)
TCCTGACCAGCTCTGCTACTGtttCCTCAGTTT  +1 (1/13)
TCCTGACCAaC------------CCTCAGTTTC  -12 (1/13)
TCCTGACCAGCTCTGC-------CCTCAGTTTC  -7 (2/13)
TCCTGACCAGCTCTGCTAC-------CAGTTTC  -7 (1/13)
TCCTGACCAGCTCTGCTACT------CAGTTTC  -6 (1/13)
TCCTGACCAGCTCTGCTACT-GCCCTCAGTTTC  -1 (2/13)
```

KKH-*tyr-T2* (14/15)

```
ATAAGAAAGGAGATATTCCGAATGAGCAGTGCT  WT (1/15)
ATAAGAAAGGAGAT--------------GTGCT  -14 (1/15)
ATAAGAAAGGA------------GAGCAGTGCT  -12 (1/15)
ATAAGAAAGGAGATATTCC----GAGCAGTGCT  -4 (12/15)
```

KKH-*tbx5-T1* (13/15)

```
CCTACTTCTCCTCAGGCTGCATTTACCCAGCAG  WT (2/15)
CCTACTTCTCCTGcAGGCTGCATTTACCCAGCA  +1 (1/15)
G--------------------------------  -169 (1/15)
CCTAC-----------------------AGCAG  -23 (1/15)
CCTACTTCT---------GCATTTACCCAGCAG  -9 (1/15)
CCTACTTC--------AGGCTGCATTTACCCAG  -8 (1/15)
CCTACTTCTCCT------GCATTTACCCAGCAG  -6 (6/15)
CCTACTTCTC---AGGCTGCATTTACCCAGCAG  -3 (1/15)
CCTACTTCTCC--AGGCTGCATTTACCCAGCAG  -2 (1/15)
```

KKH-*tbx5-T2* (11/15)

```
GAGAGAACTGTGGCTGAAATTTCACGAAGTTGG WT (4/15)
GAGAGAACTGTGGCTGAAAT--CACGAAGTTGG -2 (2/15)
--------------------------ATTATTA -54 (1/15)
GAGAGAACTGTGaCT-------------GTTGG -13 (1/15)
GAGAGAACTCT-----------CACGAAGTTGG -11 (1/15)
GAGAGAACTGTGGCT----------GAAGTTGG -10 (1/15)
GAGAGAACctc----------TCACGAAGTTGG -10 (1/15)
GAGAGAACTGTGGCTGAAATT-CACGAAGTTGG -1 (1/15)
GAGAGAcCcGTGGCTGAAATTTCACGAAGTTGG M2 (1/15)
GAGAGAACTGTGGCTGAAATTggCACGAAGTTG +1 (1/15)
GAGAGAACTGTGttggcacagaagttggcacaG +7 (1/15)
```

Fig. S9. Sanger DNA sequencing data show the mutations induced by SaCas9 and KKH SaCas9 targeting the genes showed in Fig. 5. For all the panels, the wild-type sequence is shown at the top with the target site highlighted in yellow and the PAM sequence in blue text. Red dashes indicate deletions and lowercase letters in red indicate insertions or mutations. The numbers in parentheses indicate ratio of this sequence in the total colonies sequenced. For target site designation, the gene name is in the middle, the prefixes Sa- and KKH- represent SaCas9 and KKH SaCas9, respectively. The suffixes -T1 and -T2 represent the first and the second target site designed in a given gene, respectively.

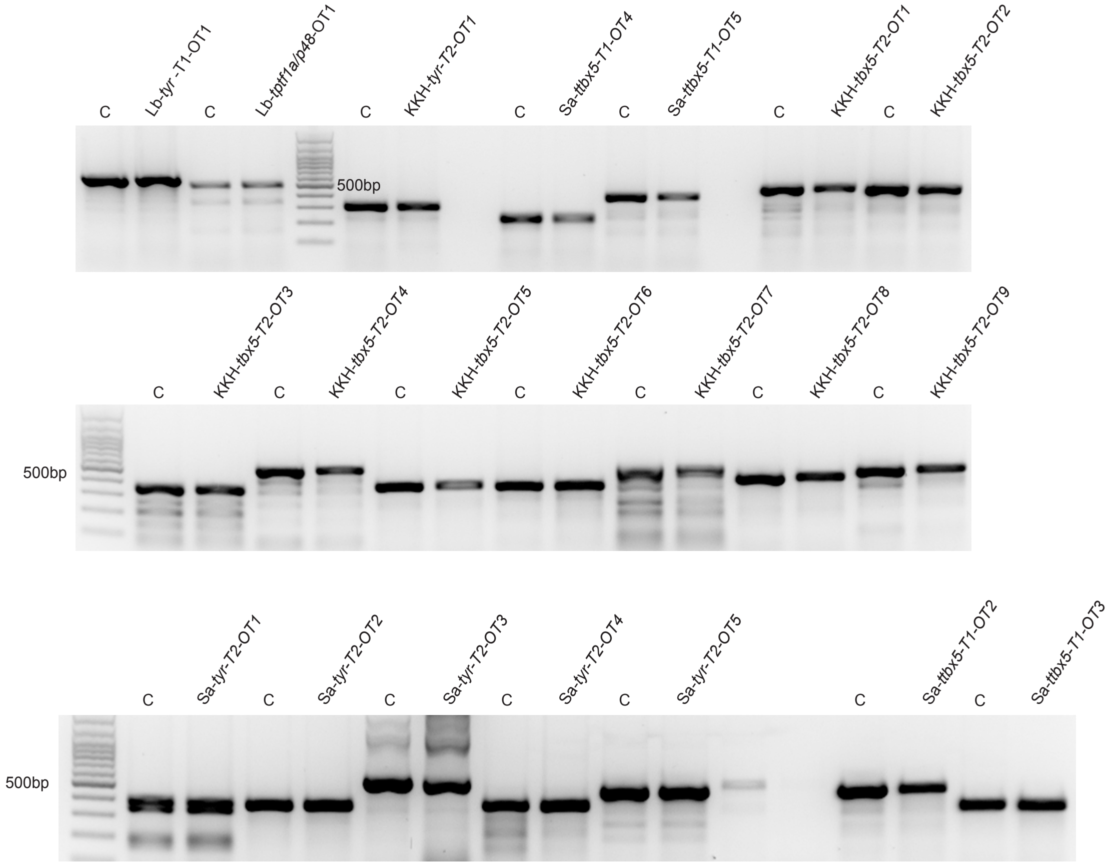

Fig. S10. T7EI assay data show no discernible mutagenic activities on potential off-target sites for LbCas12a, SaCas9, and KKH SaCas9 in *X. tropicalis* embryos. Potential off-target (OT) site is indicated as the last suffix. C, control.
